# The role of a parasite-specific D-site in activation of Plasmodium falciparum cGMP-dependent protein kinase

**DOI:** 10.1186/2050-6511-16-S1-A51

**Published:** 2015-09-02

**Authors:** Eugen Franz, Jeong Joo Kim, Olga Schneider, Daniela Bertinetti, Choel Kim, Friedrich W Herberg

**Affiliations:** 1 Department of Biochemistry, University of University, 34132 Kassel, Germany; 2 Department of Pharmacology, Baylor College of Medicine, One Baylor Plaza, Houston, TX, USA

## Background

Malaria is one of the most dangerous tropical diseases worldwide, resulting in approximately 1.5-2.7 million deaths per year [1]. Furthermore, malaria belongs to the four major infectious diseases also including HIV, tuberculosis and hepatitis. In humans, malaria is transmitted by four species of the genus *Plasmodium*. However, most malaria deaths are caused by *Plasmodium falciparum *[2].

The *Plasmodium falciparum* cGMP-dependent protein kinase (PfPKG) is one of the key regulators of the malaria parasite life cycle in both sexual and asexual blood-stages. Inhibition of PfPKG stops differentiation and transmission of the parasites, indicating that this kinase is a promising drug target for malaria [3-5]. However, despite its physiological importance, the activation mechanism of PfPKG is not fully understood. Recently, our group discovered that disrupting cGMP binding at the C-terminal cyclic nucleotide-binding (CNB-D) domain almost completely abolishes the kinase activation [6]. Therefore, we investigate the functional role of the PfCNB-D in PfPKG activation.

## Results

To investigate the role of the CNB-D in PfPKG activation, we generated a deletion mutant without the N-terminal 400 amino acid residues (Δ400), which is missing a potential auto-inhibitory sequence. We expressed the deletion construct (PfPKG 401-853) containing the catalytic domain and the PfCNB-D in *E. coli*. For the cGMP binding studies we used fluorescence polarization (FP) assay and for the activation studies we used microfluidic mobility-shift assay (MSA).

The deletion construct showed similar affinity for cGMP (40 nM) like the isolated PfCNB-D. In activation, the Δ400 mutant showed an approximately 3 fold increased activation constant for cGMP (K_A_ about 240 nM) in comparison to the full length PfPKG (KA about 70 nM [6]). Unexpectedly, the deletion mutant exhibited a very low basal activity (about 0.3 U/mg) in the absence of cGMP. However, it could be activated efficiently by cGMP (7.0 U/mg), suggesting that the allosteric activation mechanism of PfPKG is dependent only on cGMP binding.

In addition, disrupting cGMP binding through mutating the conserved arginine 492 to lysine (R492K) abolished the cGMP-dependent activation of the deletion mutant (500-600 fold higher K_A_ for cGMP), suggesting that the cGMP binding at the CNB-D mainly triggers the activation of this mutant.

## Conclusion

Our results revealed that the activation mechanism of PfPKG differ from mammalian PKGs. Without the auto-inhibitory sequence, we expected that the mutant would completely lose its control on the activity, since the catalytic cleft of the kinase is open. However, without cGMP the deletion construct (PfPKG 401-853) has a low basal activity and can be activated efficiently by cGMP. This suggests that controlling PfPKG activity may not depend on the interaction between the inhibitory sequence and the catalytic domain. Upon cGMP binding, conformational changes in the regulatory (R) domain, especially in the area that bridges the regulatory and catalytic domains, induce kinase activation.
